# Effects of Glucocorticoid Exposure on Growth and Structural Maturation of the Heart of the Preterm Piglet

**DOI:** 10.1371/journal.pone.0093407

**Published:** 2014-03-27

**Authors:** Min Young Kim, Yvonne A. Eiby, Eugenie R. Lumbers, Layne L. Wright, Karen J. Gibson, Amanda C. Barnett, Barbara E. Lingwood

**Affiliations:** 1 The University of Queensland, UQ Centre for Clinical Research, Brisbane, Australia; 2 Department of Physiology, School of Medical Sciences, University of New South Wales, Sydney, Australia; 3 School of Biomedical Sciences and Pharmacy, University of Newcastle, Newcastle, Australia; Temple University, United States of America

## Abstract

Inadequate maintenance of systemic blood flow in neonates following preterm birth is associated with increased morbidity and mortality, and may be due in part to structural immaturity of the myocardium. Maternal glucocorticoid administration is associated with improved cardiovascular function, and possibly promotes structural maturation of the myocardium. This study assessed the structural maturity of the myocardium in male and female preterm and term piglets, and preterm piglets exposed to a regimen of maternal glucocorticoids as used clinically. In preterm, term and glucocorticoid exposed preterm piglets cardiomyocyte maturity was examined by measuring the proportion of binucleated myocytes and the volumes of single living ventricular cardiomyocytes with fluorescence microscopy. Ventricular apoptosis and proliferation were measured by immunohistochemistry. Preterm piglet hearts had fewer binucleated myocytes, smaller myocytes, and more proliferative and fewer apoptotic nuclei than term hearts. Maternal glucocorticoid treatment resulted in increased binucleation with no increase in myocyte volume, and levels of proliferation and apoptosis that were more similar to the term heart. Atrial weights were increased and in female piglets there was an increase in the ratio of left to right ventricular weight. The observed changes in atrial mass and myocyte structural maturation correlated with changes in cardiac function of isolated hearts of littermates. In conclusion, the association between increased myocardial maturation following glucocorticoid exposure, improved cardiac function in littermates, and clinical improvement in human neonatal cardiac function exposed to antenatal glucocorticoids, suggests that glucocorticoid exposure contributes to improved cardiovascular function in preterm infants by promoting myocardial structural maturity.

## Introduction

The preterm neonate often fails to maintain systemic blood flow and tissue perfusion, and this is associated with increased risk of poor outcomes [1], [2], [3]. The reasons why preterm infants are unable to maintain systemic blood flow are unknown. Before more effective treatments for preterm infants can be developed, it is essential to better understand the factors that contribute to their poor cardiovascular function. Immaturity of the myocardium may be one of these factors.

Glucocorticoids are routinely administered to women threatening to deliver prematurely, and have been shown to significantly reduce morbidity and mortality, primarily due to improved lung maturation [4], [5]. Glucocorticoid exposure before birth also reduces the incidence of low systemic flow in human infants [6] and the need for blood pressure support [7], and is associated with increased aortic flow in the preterm piglet heart [8]. These changes probably contribute to the improved outcome of infants exposed to glucocorticoid antenatally. If we can identify the aspects of preterm heart growth and myocyte structure that are improved by antenatal glucocorticoid exposure, this may help to identify the factors that contribute to poor preterm function.

In late gestation or the early postnatal period cardiac myocytes lose the ability to proliferate readily and become terminally differentiated [9], [10], [11], [12], [13]. Terminal differentiation represents a shift in the mode of cell growth from hyperplasia to hypertrophy. It is morphologically marked by the cessation of cytokinesis with persistent karyokinesis resulting in single cardiac myocytes with multiple nuclei [10], [12], [14]. Terminally differentiated cardiac myocytes exhibit structural properties that may impact on function, including organized myofibrils and mitochondria, smaller nuclei, larger cell volumes and multi-nucleation [15], [16]. The increase in myocyte volume seen in the early postnatal period is thought to be due to increases in myofibrillar and cytoskeletal components as well as other intracellular structures [12], [17], [18], and it has been suggested that this increased volume of organized myofibrils physically prevents cytokinesis, resulting in binucleation and terminal differentiation [12], [17]. Although no study has directly investigated the relationship between terminal differentiation/structural maturation and contractility of cardiac myocytes, it seems likely that the smaller uninucleated myocytes of the preterm heart contain a lesser volume of myofibrils which are less organized, reducing the contractile strength of the myocyte and contributing to poor cardiac function.

Long-term, postnatal administration of glucocorticoid to the preterm infant with chronic lung disease has been associated with left ventricular hypertrophy [18], [19], [20]. However, little is known about the effects of antenatal maternal glucocorticoid treatment on cardiac maturation. Nor is it known whether or not any glucocorticoid induced maturational changes contribute to improved cardiovascular function in the preterm offspring. Maternally administered glucocorticoids may also have different effects in male and female preterm infants. Females may gain greater benefit from maternal glucocorticoid treatment than males [21]. This could contribute to the lower mortality and morbidity of both preterm human female infants and female lambs [22], [23], [24], [25].

The aim of this study was to evaluate the effects of maternally administered glucocorticoids on the structural features of the preterm piglet myocardium, including whole heart and ventricular weight, nucleation and size of cardiac myocytes, sarcomere length, and the levels of cardiac ventricular proliferation and apoptosis. We also investigated if these changes were sex specific.

## Methods

### Ethics Statement

This study was carried out in strict accordance with the recommendations in the Guide for the Care and Use of Laboratory Animals of the National Institutes of Health. The protocol was approved by The University of Queensland Animal Ethics Committee (AEC Approval Number: UQCCR/999/08) All surgery was performed under isoflurane or propofol anaesthesia, and all efforts were made to minimize suffering.

### Animals

Large White X Landrace piglets were delivered by caesarean section at two ages, preterm piglets delivered at 91 days gestation (term - 115 days) and term piglets delivered two days before the expected farrowing date. At 91 days, preterm piglets are approximately half of term weight, have very thin translucent skin, thermoregulate very poorly and require similar respiratory and cardiovascular support to a baby born at 25–27 weeks gestation [26]. An additional group of preterm piglets was exposed to maternally administered glucocorticoids (betamethasone, 0.19 mg/kg body weight, given i.m.; Celestone Chronodose; Schering-Plough, USA) given 48 h and 24 h before delivery. The timing and dose/kg are equivalent to that given to women presenting with threatened preterm labour. In each of the three treatment groups, three litters of piglets were studied. Four piglets (similar sex ratios) from each litter were randomly assigned to this experiment and four littermates were randomly assigned to another experiment that investigated cardiac function [8]. Piglets with a birth weight below the 10^th^ percentile were excluded from both studies.

### Surgery

Pregnant sows (280–350 kg) were premedicated with 400 mg azaperone given i.m. (Stresnil; Janssen, Australia). Anaesthesia was induced with 200 mg of alfaxalone given i.v. (Alfaxan-CD RTU; Jurox, Australia), followed by administration of additional alfaxalone as required to allow intubation of the trachea. The total administered dose of alfaxalone was 300–700 mg. Anaesthesia was maintained with 2% isoflurane (Attane Isoflurane USP; Minrad, USA) in O_2_ and sows breathed spontaneously. Throughout surgery (approximately 2 h), saline (2-3L of 0.15M NaCl) was administered via an ear vein and the following variables were monitored: arterial blood pressure in the tail by Doppler (Parks Medical Electronics Inc, Aloha, OR, USA), O_2_ saturation by pulse oximetry (Masimo, Irvine, CA, USA), end tidal isoflurane and end tidal P_CO2_ concentrations (Capnomac Anaesthesia Monitor, Datex-Ohmeda Inc, Madison, WI, USA).

Caesarean delivery was performed via a ventral midline incision. Following incision into the linea alba the uterus was exposed. Piglets were individually removed from the uterus at approximately 10 min intervals, anaesthetised with approximately 5 mg/kg propofol (Provive 1%; AFT Pharmaceuticals, New Zealand) via the umbilical vein and weighed and sexed. The piglet's chest was opened and the heart was rapidly excised and placed into modified Krebs solution (125 mM NaCl, 4.75 mM KCl, 1.2 mM KH_2_PO_4_, 1.2 mM MgSO_4_, 20 mM HEPES, 10 mM BDM and 5.5 mM glucose; pH 7.4). After all the piglets were delivered the sow was euthanized by IV injection of pentobarbital sodium (65 mg/kg, Lethabarb, Virbac, Australia).

### Cardiac Myocyte Characteristics

With development, the volume of individual cardiac myocytes increases as does the number of terminally differentiated (i.e., multinucleated) myocytes [9], [13], [27]. These features were used as markers of myocardial maturation and were assessed in freshly isolated, live myocytes to avoid artefactual alterations in cell volume associated with fixation.

#### Cardiac Myocyte Isolation

Fresh piglet cardiac myocytes were isolated using methods described previously with minor modifications [9], [28]. Briefly, excised hearts were weighed and the free walls of the left and right ventricles, the atria, and the interventricular septum were dissected and individually weighed. A strip of myocardium (approximately 0.5×1.5 cm) extending from epicardium to endocardium and from the atrioventricular groove to the apex, in the centre of each ventricular free wall, was removed and finely chopped.

Single cardiac myocytes were released by enzymatic digestion (Blenzyme 3, 0.339Wűnsch units/ml, Roche, Germany) in modified Krebs solution (composition described above) for 2 h at 37°C. The enzyme solution was replaced every 30 min to discard cell debris. Digested tissue blocks were washed twice in modified calcium-free relaxing solution (40 mM KCl, 80 mM NaCl, 20 mM HEPES, 5 mM K_2_EDTA, 5.5 mM glucose; pH 7.4) to prevent myocyte contraction, followed by incubation at room temperature for 30 min following each wash. Release of single fresh cardiac myocytes was accelerated with trituration at regular intervals throughout the digestion and wash steps. At the end of the wash steps, the solution above the tissue blocks was transferred to new tubes and centrifuged at 180×g for 1 min at room temperature. The pellets were dispersed in 500 μl of fresh relaxing solution. Ethidium bromide (ICN Biochemicals Inc., Costa Mesa, CA) was added (final concentration of 20 μg/ml) immediately prior to microscopic examination.

#### Cardiac Myocyte Nucleation and Volume

Cardiac myocyte nucleation and volume were determined as previously described [9], [27], [28]. Damaged or super-contracted cells were excluded. The cells were examined with a Zeiss fluorescence microscope (Axio Imager M1, Zeiss EC Plan-NEOFLUAR 40×/0.75, the Carl Zeiss Group, Germany) with FITC absorbance setting. To determine the proportions of uni- and binucleated cardiac myocytes in each ventricle from each piglet, 100–200 cells per ventricle were examined in each animal as previously described [9]. The ratio of binucleated to total number of myocytes was calculated for each ventricle.

To measure the volumes of individual cardiac myocytes, live, isolated cardiac myocytes were examined using a laser scanning fluorescence confocal microscope (Zeiss LSM710, Zeiss Plan Apochromat 63×/1.40 Oil DIC M27, the Carl Zeiss Group, Germany) equipped with HBO 100 argon ion laser, capable of excitation at multiple wavelengths. The smart setup excitation/absorbance wavelength pre-set for ethidium bromide (LSM software Zen 2008 software, Carl Zeiss Group, Germany) was selected (excitation wavelength at 514 nm, detection wavelength range from 552–693 nm with a detection filter with pinhole width of 37 μm).

Methods for determining the volumes of live myocytes using ImageJ software (National Institute of Health, http://rsbweb.nih.gov/ij/) have been described previously [9], [27], [28]. The number of myocytes assessed for each ventricle in each animal ranged from 9–18 for uninucleated myocytes and from 5–14 for binucleated myocytes in term animals only. For each individual myocyte a Z-series of up to fifteen 1.5 μm thick optical sections was scanned and saved for later analysis. Cross sectional areas from each optical section of a cardiac myocyte and the volume of individual cardiomyocytes were calculated using a formula previously described [9], [28], [29].

#### Myocardial Cell Proliferation and Apoptosis

A strip of myocardium (approximately 0.5×1.5 cm) extending from epicardium to endocardium and from the atrioventricular groove to the apex, in a central location and adjacent to that used for myocyte isolation, was fixed in 10% neutral paraformaldehyde overnight and dehydrated in 100% alcohol. Tissue strips were embedded in paraffin, sliced into 3.0–4.0 μm sections and dewaxed. Endogenous peroxidase activity was inhibited (1% H_2_O_2_, 0.1% sodium azide in PBS for 10 min) and the background was blocked separately for each immunostain (caspase-3 - 20 min in 10% normal goat serum; Ki-67 - 4% skim milk in PBS for 20 min followed by 10% donkey serum for 30 min). Tissue sections were then immunostained against caspase-3 (apoptotic marker; a polyclonal rabbit antibody specific for large fragments (17–19 KDa) of cleaved caspase-3, 1∶120, Biocare Medical, ASP175, CP229) or Ki-67 (proliferation marker; monoclonal mouse anti-human Ki-67, 1∶90 in Vector 10% donkey serum, Dako, M7240 and Impress HRP-conjugated secondary anti-mouse, Vector Labs MP-7401). The immunostained sections were visualised using horseradish peroxidase (HRP) conjugated DAKO Envision plus reagent (cat no. K4003, anti-rabbit), using diaminobenzidine (DAB) as the chromogen. Sections were lightly counterstained in Mayers' haematoxylin, dehydrated, cleared and mounted in DePeX.

To control for the subjectivity inherent in a manual scoring process, the quantitative analysis of the caspase-3 and Ki-67 immunohistochemical staining was largely automated using the Aperio Scanscope system. The immunolabelled slides were scanned at ×20 magnification (resolution 0.499 μm/pixel) using the Scanscope XT slide scanner (Aperio Technologies, Vista, CA, USA). The acquired digital images of entire tissue sections were viewed at high resolution using ImageScope viewer software (V10.2). An independent, trained observer, blind to experimental treatment conditions, used a preset-sized rectangle annotation tool (225 μm^2^) to select 12 areas of tissue in three zones: rostral, middle and caudal. Within each of these zones 1 inner, 1 outer and 2 mid-sectional areas were selected. The annotations were placed to avoid large blood vessels and tissue and staining artefacts. The annotations were assessed using the Aperio IHC nuclear Algorithm (V10) designed to measure the number and intensity of positively stained nuclei based on set parameters. A mean of 1700 nuclei per ventricle were assessed.

#### Sarcomere Length

Sarcomere length was assessed in 3.0–4.0 μm sections of fixed myocardium stained with haematoxylin and eosin. Z-bands were counted within 10 μm long segments of myocytes. Three measurements were taken in each section and averaged [30]. No correction was applied for preparation shrinkage.

### Data analysis and statistics

Data were analysed with the statistical software program IBM SPSS v20 (SPSS Inc., Chicago, USA). Binucleation data were transformed using arcsine square root function. Effects of treatment group, sex and their interaction, on body and heart weights, binucleation, volumes, Ki-67 and Caspase-3 labelling were detected using 3-way ANOVAs (with group and sex as fixed factors and litter as a random factor nested in group). Significant differences were reported only where these existed independently of litter effects. Where differences between piglet groups were detected, post hoc tests (Fisher's least significant difference) were used to identify where these differences lay. Where interactions between group and sex were detected, posthoc tests (Tukey HSD) were used to identify where these lay. Paired t-tests were used to compare left and right ventricular parameters, and the volume of bi- and uninucleated myocytes in left and right ventricles at term. Sarcomere length was not normally distributed after transformation and so Kruskal-Wallis tests were used to assess differences between groups for each ventricle. Then, within each group, Mann Whitney tests were used to assess sex differences for each ventricle and Wilcoxon signed rank tests compared left and right ventricles. Statistical significance was set at *P*<0.05.

## Results

### Animal groups

In each of 3 groups (term, preterm and preterm + glucocorticoid), 12 piglets (6 males, 6 females) were studied.

### Effects of Glucocorticoids on Heart and Body Weight

To determine whether glucocorticoid exposure alters growth of the heart or whole body, heart and body weights in term, untreated preterm and glucocorticoid exposed preterm piglets were compared. Details of body and heart weight, and weights of heart components are shown in Table 1. Preterm piglets were approximately half the weight of term piglets (*P*<0.001) and there were no significant differences in body weight between male and female piglets in any group (Table 1). There was a significant interaction between group and sex (*P* = 0.050) such that the body weight of glucocorticoid exposed male preterm piglets was not different from untreated preterm piglets (*P* = 0.605), but glucocorticoid exposed female preterm piglets were heavier than their untreated counterparts (*P* = 0.022) (Table 1).

**Table 1:** Body weight, heart weight and weight of components of the heart as absolute value and percentage of body weight in male and female piglets.

Parameter		Preterm 91d	Preterm 91d + GC	Term 113d
		Male *n* = 6	Male *n* = 6	Male *n* = 6
		Female *n* = 6	Female *n* = 6	Female *n* = 6
Body weight (g)*^δ^	*Male*	750±72	701±50	1329±126
	*Female*	569±50	801±46	1384±101
Heart weight (g)*	*Male*	4.90±0.48	4.60±0.15	9.62±0.78
	*Female*	3.84±0.35	5.14±0.33	9.54±0.53
Heart: Body (%)*	*Male*	0.653±0.022	0.665±0.027	0.729±0.018
	*Female*	0.675±0.023	0.640±0.016	0.694±0.014
Ventricular weight (g)*	*Male*	3.46±0.31	2.91±0.11	6.54±0.54
	*Female*	2.71±0.34	3.37±0.35	6.64±0.38
LVFW weight (g)*^δ^	*Male*	1.32±0.14	1.10±0.03	2.32±0.19
	*Female*	0.89±0.06	1.32±0.09	2.55±0.16
RVFW weight (g)*	*Male*	1.20±0.13	1.04± 0.056	2.53±0.21
	*Female*	1.09±0.19	1.04±0.10	2.38±0.19
LVFW:RVFW^δ^	*Male*	1.12±0.08	1.07±0.06	0.92±0.04
	*Female*	0.91±0.11	1.32±0.10	1.10±0.08
IVS weight (g)*	*Male*	0.94±0.13	0.77±0.05	1.69±0.15
	*Female*	0.74±0.10	1.02±0.21	1.71±0.12
Atria weight (g)*^β^	*Male*	1.44±0.23	1.68±0.047	3.08±0.27
	*Female*	1.13±0.09	1.77±0.21	2.90±0.22
Ventricular: Body (%)	*Male*	0.467±0.032	0.421±0.016	0.496±0.014
	*Female*	0.471±0.027	0.416±0.022	0.483±0.014
LVFW: Body (%)	*Male*	0.178±0.013	0.160±0.011	0.177±0.009
	*Female*	0.158±0.010	0.165±0.005	0.187±0.011
RVFW: Body (%)^δ^	*Male*	0.161±0.010	0.149±0.004	0.192±0.003
	*Female*	0.185±0.020	0.128±0.008	0.172±0.005

Data expressed as mean ± SEM, GC  =  glucocorticoid exposed, LVFW  =  left ventricular free wall, RVFW  =  right ventricular free wall, IVS  =  interventricular septum. *Preterm group is different to the term group (*P*<0.05). ^β^Glucocorticoid exposed preterm group is different to untreated preterm group (*P*<0.05). ^δ^Glucocorticoid exposed preterm group is different to untreated preterm group for females but not males (*P*<0.05).

The heart weights of preterm piglets were also about half of those of term piglets (*P*<0.001) and there were no significant differences in heart weight between male and female piglets in any group (Table 1). The heart weights of glucocorticoid exposed preterm piglets were not different from those of untreated preterm piglets (Table 1). Expressed as a percentage of body weight, the heart weights of all 3 groups of animals were similar (Table 1) and there were no differences between sexes. Most heart component weights reflected these patterns with all weights being lower in preterm piglets than in term piglets and no differences between male and female piglets. Atrial weights were greater in glucocorticoid exposed preterm piglets than in untreated preterm piglets (*P* = 0.017) (Table 1). There were significant interactions between group and sex for left ventricular weight (*P* = 0.016), right ventricular weight as a proportion of body weight (*P* = 0.005) and the ratio of left to right ventricular weight (*P* = 0.043). Female glucocorticoid exposed preterm piglets had increased left ventricular weight (*P* = 0.018), decreased right ventricular weight as a proportion of body weight (*P*<0.001) and increased ratio of left to right ventricular weight (*P* = 0.001) compared to untreated female preterm piglets. There was no effect of glucocorticoid exposure in male preterm piglets (Table 1).

### Effects of Glucocorticoids on Preterm Myocyte Maturity

The structural maturation of the preterm heart was assessed in terms of the proportion of binucleated myocytes, the volume of myocytes, sarcomere length, and the number of proliferative and apoptotic nuclei.

#### Proportion of binucleated myocytes

For this and all following parameters, there was no difference between male and female piglets in any group (LV and RV: P>0.1) and no interaction between sex and group (LV and RV: P>0.1). Therefore results from male and female piglets were combined.

Only two myocytes were found that had more than two nuclei. These two multinucleated cells were included in the population of binucleated myocytes. One multinucleated myocyte was from the right ventricle of a female glucocorticoid treated preterm piglet and the other was from the left ventricle of a female term piglet.

**Figure 1:**
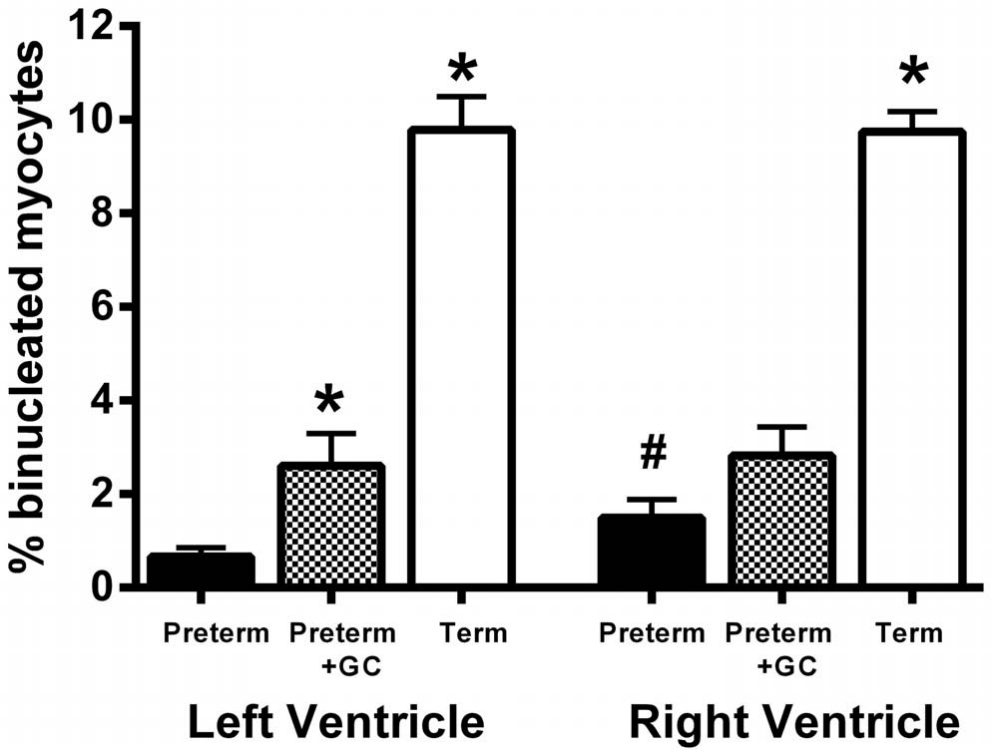
% Binucleated Myocytes. The proportion of cardiac myocytes that were binucleated in the left and right ventricle of untreated preterm (solid bar), preterm + GC (glucocorticoid exposed, dotted bar) and term (open bar) piglet hearts. Mean ± SEM. N = 12 for all groups. * *P*<0.05 compared to untreated preterm group. ^#^ *P*<0.05 compared to left ventricle within the same group (paired t-test). Significant differences indicated only where these existed independently of litter effects.

In both left and right ventricles the proportion of cardiac myocytes that were binucleated was significantly lower in preterm compared to term piglets (*P*<0.001) (Fig 1). Maternal glucocorticoid treatment was associated with an increased proportion of binucleated cardiac myocytes in the left ventricle (*P* = 0.025) with a similar trend in the right ventricle (*P* = 0.092) (Fig 1). The proportion of myocytes that were binucleated was higher in the right ventricle than in the left ventricle in untreated preterm piglets (*P* = 0.017) but not in glucocorticoid treated preterm piglets or term piglets (*P* = 0.79 and 0.94) (Fig 1).

#### Volume of myocytes

Myocyte volume was measured in a total of 325 myocytes from preterm piglets, 328 myocytes from glucocorticoid exposed preterm piglets and 452 (282 uninucleated and 170 binucleated) from term piglets. Because there were so few binucleated myocytes in preterm and glucocorticoid exposed animals, only uninucleated myocytes were compared in these groups. Binucleated myocytes were larger than uninucleated myocytes at term in both the left (P<0.001) and right (P<0.001) ventricles (Table 2).

**Table 2:** Volume of uninucleated and binucleated cardiac myocytes in term piglets (μm^3^).

Myocyte Volume (μm^3^)	Uninucleated myocytes	Binucleated myocytes
Left Ventricle N = 12	2459±150	3341±308^§^
Right Ventricle N = 12	3111±219^#^	4372±332^#§^

Data are mean ± SEM of average volume of 5–18 myocytes per ventricle in each pig. ^#^ *P = *0.058 for uninucleated and 0.050 for binucleated compared to left ventricle within the same animal (paired t-test). ^§^ *P*<0.001 compared to the uninucleated within the same ventricle (paired t-test).

**Figure 2:**
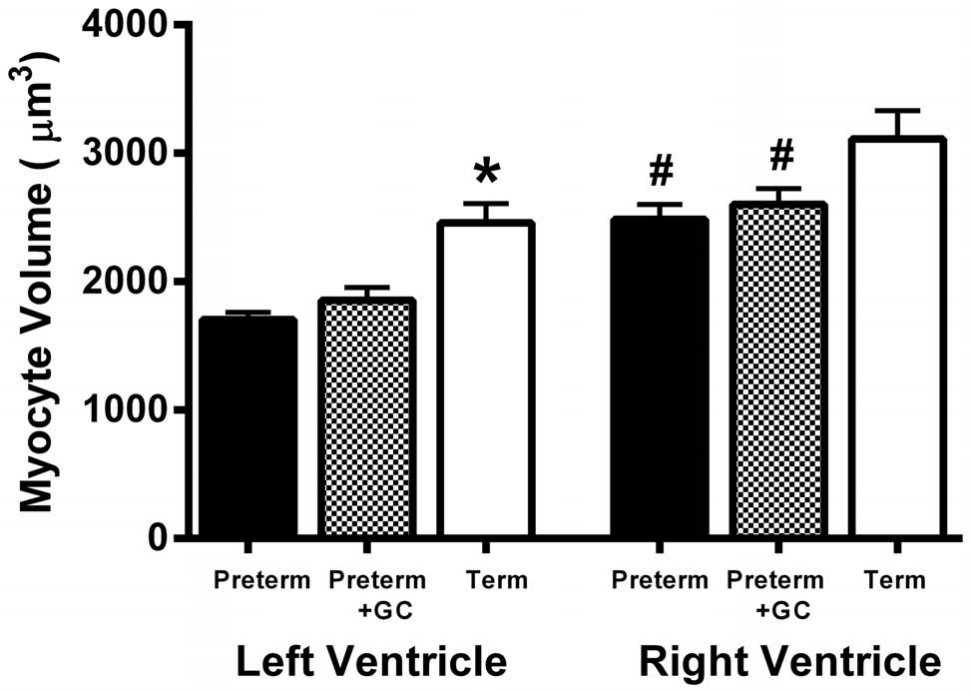
Myocyte Volume. The volume of uninucleated myocytes in the left and right ventricle of untreated preterm (solid bar), preterm + GC (glucocorticoid exposed, dotted bar) and term (open bar) piglet hearts. Mean ± SEM. N = 12 for all groups. * *P*<0.05 compared to untreated preterm group. ^#^ *P*<0.05 compared to left ventricle within the same group (paired t-test). Significant differences indicated only where these existed independently of litter effects.

In the left ventricle uninucleated myocytes from preterm piglets were smaller than uninucleated myocytes from term piglets (*P*<0.001), but in the right ventricle there was no difference between myocyte volumes in preterm and term piglets hearts (*P = *0.081) (Fig 2). Myocyte volume was not altered by maternal glucocorticoid treatment in either ventricle (LV: *P = *0.388, RV: *P = *0.081). In untreated and glucocorticoid exposed preterm piglets, uninucleated myocytes from the right ventricle were larger than those from the left ventricle (*P*<0.005) (Fig 2). The same pattern was present at term but did not quite reach significance (*P = *0.058 for uninucleated and 0.050 for binucleated myocytes, Table 2).

#### Sarcomere Length

**Figure 3:**
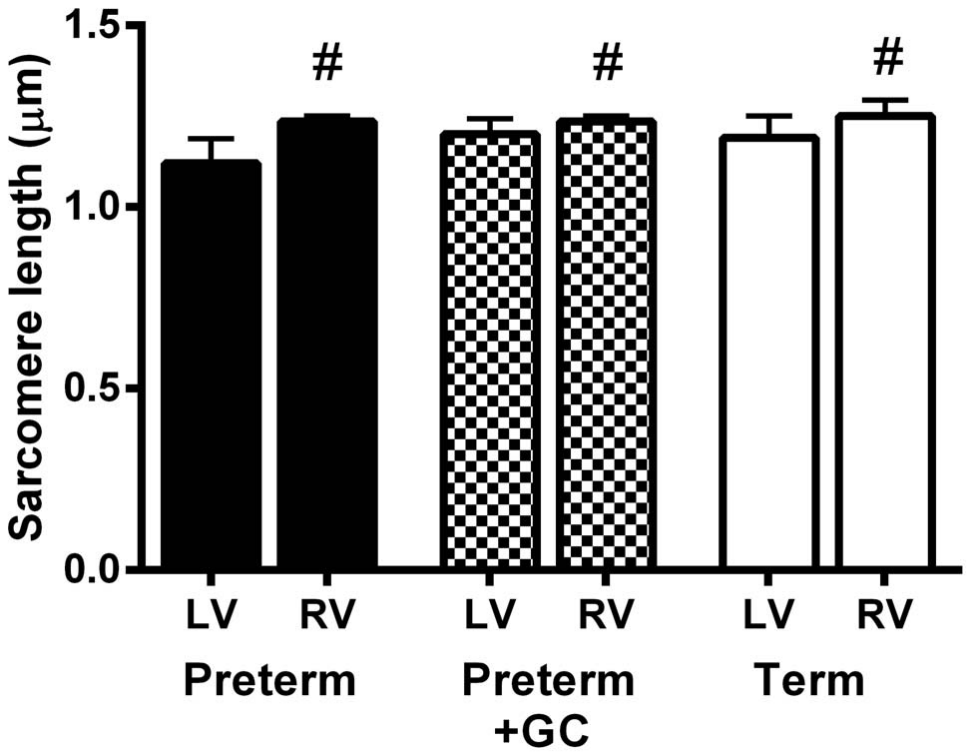
Sarcomere length. Sarcomere length in the left (LV) and right ventricle (RV) of untreated preterm (solid bar), preterm + GC (glucocorticoid exposed, dotted bar) and term (open bar) piglet hearts. Mean ± SEM. N = 12 for all groups except N = 8 for term LV. ^#^ *P*<0.05 compared to left ventricle within the same group (Wilcoxon signed rank test).

In both left and right ventricles, sarcomere length was not affected by gestational age or glucocorticoid exposure (left, P = 0.146; right, P = 0.381). Sarcomere length was increased in the right ventricle compared to the left ventricle in all groups (untreated preterm, P = 0.018; treated preterm, P = 0.010; term, P = 0.012) (Fig 3).

#### Proliferation and Apoptosis

**Figure 4:**
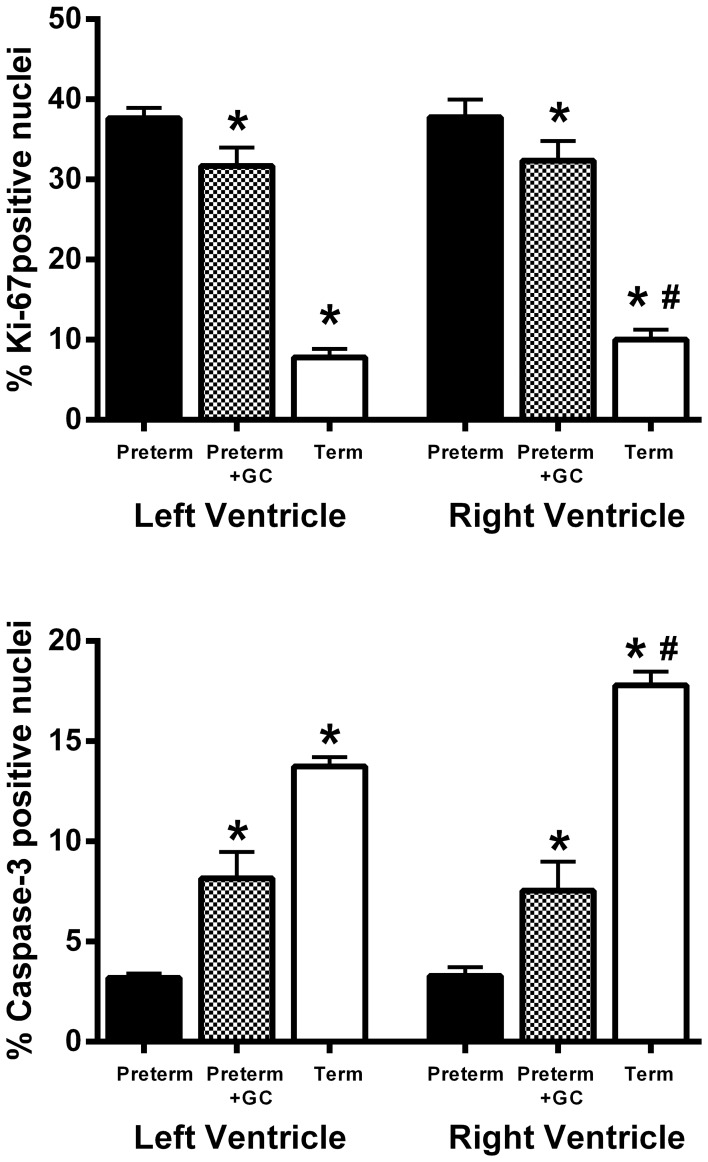
Proliferation and Apoptosis. The % of proliferating (Ki-67 stained – upper panel) and apoptotic (Caspase-3 stained – lower panel) nuclei in the left and right ventricle of untreated preterm (solid bar), preterm + GC (glucocorticoid exposed, dotted bar) and term (open bar) piglet hearts. Mean ± SEM. N = 12 for all groups. * *P*<0.05 compared to untreated preterm group. ^#^ *P*<0.05 compared to left ventricle within the same group (paired t-test). Significant differences indicated only where these existed independently of litter effects.

Staining of the myocardium with Ki-67 showed that in both left and right ventricles cell proliferation was greater in preterm hearts compared to term hearts (*P*<0.001) (Fig 4A). Proliferation was lower in glucocorticoid exposed preterm hearts compared to untreated preterm hearts (*P* = 0.006) (Fig 4A). At term the % positive Ki-67 nuclei was higher in the right ventricle than in the left ventricle (*P* = 0.031, Fig 4A).

Staining of the myocardium with Caspase-3 showed that in both left and right ventricles apoptosis was lower in preterm hearts compared to term hearts (*P*<0.001) (Fig 4B). Apoptosis was increased in GC exposed preterm hearts compared to untreated preterm hearts (*P*<0.001) (Fig 4B). At term the % positive caspase-3 nuclei was higher in the right ventricle than in the left ventricle (*P* = 0.05, Fig 4B).

## Discussion

The present study describes the morphological changes in cardiac myocytes in piglet heart in late gestation, and the effect of maternal glucocorticoid treatment on these. Left and right ventricle have been separately studied and the effects of sex were also determined. The study provides the first clear evidence that antenatal glucocorticoid exposure, at a clinically relevant dose, alters cardiac growth and development, and that this may contribute to the observed improvements in cardiovascular function following glucocorticoid exposure.

### Characteristics of the Preterm Piglet Heart

The preterm heart was smaller than the term heart but was in the same proportion to body weight as in the term animal. There were no differences in the weights of the body, heart or heart components between male and female piglets.

In preterm hearts, myocytes were smaller and there were fewer binucleated myocytes than in the term heart, indicating that there is increasing maturity of the piglet heart over the study period. However, sarcomere length in the preterm heart was not different to the term heart. While it has been suggested that sarcomere length increases as the sarcomeres are assembled and the myocytes grow [31], [32], there was no difference in sarcomere length between day 1 and day 11 in neonatal rats [33]. The lack of any difference in sarcomere length between untreated preterm and term piglet hearts indicates that, in the preterm piglet, sarcomere length cannot be considered an index of cardiomyocyte maturation over this gestational age range during which other maturational changes clearly occur.

The higher levels of Ki-67 immunolabeling (a marker of cell cycle activity) and lower levels of caspase-3 immunolabeling (a marker of apoptosis) in preterm piglet hearts confirms that at 91 days gestation (equivalent to 25–27 weeks human gestation) the heart is growing by hyperplasia as well as hypertrophy, as seen in other species [12], [34], [35]. During the last weeks of pregnancy in the pig, however, there is evidence that this hyperplastic growth declines since the number of Ki-67 positive nuclei were less at term (Figure 4A), while hypertrophy continued as indicated by the increase in myocyte volume (Figure 2). Interestingly term piglet hearts had a higher degree of apoptosis, perhaps indicative of cardiac remodeling prior to transition to a neonatal circulation.

In untreated preterm piglet hearts myocytes in the right ventricle were larger than those in the left ventricle, and there were more binucleated myocytes in the right ventricle than in the left. Sarcomere length was longer in the right ventricle than in the left ventricle in both preterm and term hearts. These differences reflect the dominance of, and greater load on, the right ventricle *in utero*, and have also been reported in sheep, rodents and humans [9], [12], [13], [15]. As term approached the difference in myocyte size became less apparent in the piglet heart. Right ventricular myocytes did not significantly increase in size over the period studied while left ventricular myocytes did increase, suggesting that the pattern of growth in late gestation is focussed on preparation of the left ventricle for the increase in workload that occurs after birth.

Trends in cardiac myocyte maturation reported in rats, sheep and humans [9], [13] are similar to those in this study. Piglet uninucleated cardiac myocyte volumes were comparable to those found in sheep and rodents [9], [12], but there are differences in the maturational profiles of these species in terms of the appearance of binucleation. In both the human [13] and the piglet (Fig 1), 2–5% of myocytes are binucleated at approximately 0.6–0.8 gestation, increasing to around 10% at term. However, myocytes mature earlier in sheep so that near term approximately 70% of myocytes are binucleated [9], [35]. Thus the piglet heart may be a better model than the sheep or rat for the study of human preterm heart function, because its structural maturity is very similar to the human heart. Proliferation results are similar to those reported in rats where approximately 35% of myocytes stained positively for Ki-67 at P0 and this proportion declined with age [36], [37].

### Effects of Glucocorticoids on Preterm Heart Growth

In both male and female preterm piglets, atrial weight increased in those animals that received antenatal glucocorticoids. Doppler echocardiography studies have shown that in the neonate, ventricular filling is more dependent upon atrial contraction than in the adult [38]. Thus the glucocorticoid induced increase in atrial mass may lead to increased ventricular filling and improved cardiac function.

In female piglets, glucocorticoid exposure promoted growth of the body and left ventricle with a reduction in the relative size of the right ventricle, and an increase in the ratio of left to right ventricular weight. Previous studies of the effects of maternal glucocorticoid treatment on fetal growth report no effects of a single course in human infants [39], [40] and no effect on cardiac dimensions measured by ultrasound [41]. However none of these studies have separately analysed male and female infants. If there are different effects in male and female offspring, as suggested by our study, this information may be lost when the combined population is analysed, thus suggesting no effect, and obscuring a real sexually dimorphic effect. Animal studies showing growth restriction following glucocorticoid treatment often use higher doses than the current study, or use long term infusions [42], [43]. Studies in sheep, using a single course of glucocorticoid at a similar dose to that used in the human, show conflicting results. Quaedackers et al (2005) found no effect on birthweight in a combined sex analysis [44] while Miller et al (2012) observed reduced birth weight in both males and females with greater effects in females [45]. Another report [46] shows a dose effect on growth in males only, with no effect on birthweight in females. Likewise the current study showed no effect when the sexes were combined, but sex specific effects were present. These effects remained when a larger number of animals, including additional litters, were studied [26]. The reason for this surprising result is unclear and further investigation is required in this area. Be that as it may, we would suggest that the larger body and left ventricular size, and the greater ratio of left to right ventricular weight in females treated with antenatal glucocorticoids could contribute to the improved neonatal outcome of preterm human female infants.

### Effects of Glucocorticoids on Preterm Myocyte Maturity

Glucocorticoid exposed preterm piglet hearts had an increased proportion of binucleated myocytes. This may indicate increased myofibrillar material within the cell preventing complete cell division, but perhaps increasing the contractile force that can be generated. There was no difference in the proportion of binucleated myocytes between male and female piglet hearts. It is possible, however, that our small sample size contributed to the lack of sex effect on myocyte maturity. Although the formation of binucleated myocytes is considered an early indicator of the commencement of hypertrophic growth [10], there was no statistically significant increase in myocyte volume. After maturation is initiated, myocyte volume increases more slowly than binucleation [12]. Myocytes were studied only 48h after glucocorticoid exposure and thus there may have been insufficient time for significant increases in volume to occur. It was perhaps not surprising that glucocorticoid exposure had no effect on preterm sarcomere length, since neither left nor right ventricular sarcomere length was increased in term piglets compared with untreated preterm piglets. This measure cannot therefore be considered as a marker of myocyte maturity over this gestational age range in piglets.

Maternal glucocorticoid exposure resulted in a change in the pattern of growth of the preterm myocardium so that it was more like the term heart. That is, proliferation was reduced, suggesting a reduction in hyperplasia, while Caspase-3 staining was increased, possibly suggesting remodelling. Reduced proliferation of myocytes following glucocorticoid exposure was also reported in neonatal rats [36]. Increased Caspase-3 staining could also indicate an increase in the noncanonical Wnt protein signalling pathway that is involved in cardiomyocyte differentiation activity [47], and thus may indicate increased maturation. These changes will probably also have long term implications for heart development as an overall reduction in myocyte number might contribute to later cardiovascular disease [36], [48].

Cardiac maturational effects of glucocorticoid exposure have also been reported in other species. These effects include increased length of myocytes [49], increased cardiac growth [27], [50], decreased proliferation [36] and maturation of energy producing pathways [51]. These changes could be the result of glucocorticoid induced upregulation of angiotensinogen expression in the myocardium [27], as the renin angiotensin system is critical to cardiac development [52]. Effects on cardiac growth do not appear to be modulated through MAPK pathways as these are not altered by cortisol exposure in fetal sheep [27]. Levels of both glucocorticoid and mineralocorticoid receptors also appear to be unaltered [27]. Further investigation is required to understand the pathways leading to cardiomyocyte maturation following glucocorticoid exposure.

### Does Glucocorticoid Exposure Result in Improved Cardiac Function?

Cardiac function was studied using an isolated working heart model in littermates of the piglets included in this study. Cardiac output/kg body weight in untreated preterm hearts was ≈50% lower than that of term hearts, with a reduced ability to maintain aortic flow in the face of increasing afterload [8]. Glucocorticoid exposed preterm hearts had a greater ability to maintain aortic flow in the face of increasing afterload compared to untreated preterm piglets hearts. There were no differences between male and female piglets [8]. This improvement in cardiac function of the isolated heart indicates that some of the improvement in cardiovascular function following antenatal glucocorticoid exposure is independent of the benefits of this treatment on other organ systems e.g. lungs. Cardiac function of preterm piglet littermates in this study could not be measured *in vivo* as resuscitation is not possible at this gestation. We have however, observed more stable *in vivo* cardiovascular function in glucocorticoid exposed preterm piglets compared to untreated preterm piglets in slightly more mature piglets (97 compared with 91 days gestation) [26].

These changes in preterm cardiac function parallel results of the current study where the preterm heart showed evidence of greatly reduced maturity compared to the term heart. During perinatal development, cardiomyocyte contractile elements such as myofibrils, transverse tubules, sarcoplasmic reticulum and intercalated discs all become increasingly numerous and more organised in appearance [15], [53]. The immature preterm left ventricle with fewer binucleated myocytes and altered growth patterns will contain myocytes with a reduced amount of organised contractile material. As a result it is probably less capable of generating the strong, coordinated ventricular contraction required for mature cardiac function.

Improvements in cardiac function following glucocorticoid exposure in littermates and in other piglets also have structural parallels in the current study. The increased atrial mass and greater cardiomyocyte maturity observed following glucocorticoid exposure in this study may contribute to improved cardiac function in glucocorticoid exposed preterm piglets. If similar changes occur in the human infant, this may contribute to the reduced incidence of low systemic flow and the reduced need for blood pressure support seen in infants exposed to maternal glucocorticoid treatment [6], [7]. However our failure to find sex differences in either the structure or function of the preterm heart suggests that this aspect of development does not contribute to the different cardiovascular outcomes of male and female human infants. The next step in the development of improved treatments for poor cardiovascular function in the preterm neonate will be to investigate how glucocorticoid exposure produces increased myocyte maturation, and to determine if this can be reproduced by a targeted therapy.

### Methodological Considerations

Our study utilised established methods for the measurement of volume of cardiac myocytes [9], [27], [28]. The advantages of this technique are that single, live cardiac myocytes are freshly prepared and that the measurements are made only on viable cells. In addition, we believe that the estimation of cell volume using areas obtained from a series of optical sections of myocytes better represents the real volume of myocytes than estimation from single measurements of cell length and width. Unfortunately this methodology limits the number of cells that can be measured in each animal due to time and cell viability constraints. However, we believe that this disadvantage is outweighed by the benefits of measurements in live cells, unaffected by the alterations in volume which may be induced by fixation. This method of volume measurement of cardiac myocytes was previously validated with a high reproducibility and a coefficient of variation of 2.5% among three independent investigators [9]. The volumes obtained using this method were comparable to the calculated volume from cell length and width within the same species in an independent investigation [35].

It was not possible to assess sarcomere length in fresh myocytes because striations were often not clearly visible, especially in preterm hearts. This is consistent with previous studies reporting that contractile proteins are poorly organized in the fetal sheep and few striations are clearly visible prior to term [15], [53]. Sarcomere length was assessed in fixed sections where striations were more visible, however we cannot be certain that shrinkage due to fixation and processing is consistent across groups, especially if the amount of contractile protein, and therefore water, is altered by development as indicated by both Smolich and Rudolph [15], [53].

### Conclusions

This study describes a maturation profile in the preterm piglet heart that is similar to that in the human infant, confirming the importance of this animal model for research aiming to understand human preterm cardiovascular function. In this model, maternal glucocorticoid treatment resulted in increased atrial mass and probably greater ventricular filling, and in female piglets only, an increase in left ventricular size. In addition, myocyte maturation was increased as indicated by increased binucleation, changes in proliferation and apoptosis to be more like term hearts. The lack of sex differences in myocardial maturity suggests that differences in myocardial maturity are not responsible for the poorer outcome in male neonates. The association of increased atrial mass and myocardial maturity following glucocorticoid exposure with improved cardiac function in littermates, and a clinical improvement in cardiac function in human infants exposed to glucocorticoid treatment, suggests that glucocorticoid exposure improves cardiovascular function in preterm infants by increasing cardiac structural maturity.
